# Development of an accumulation assay and evaluation of the effects of efflux pump inhibitors on the retention of chlorhexidine digluconate in *Pseudomonas aeruginosa* and *Staphylococcus aureus*

**DOI:** 10.1186/s13104-017-2637-2

**Published:** 2017-07-26

**Authors:** Molly Mombeshora, Stanley Mukanganyama

**Affiliations:** Biomolecular Interactions Analyses Group, Department of Biochemistry, University of Zimbabwe, P.O. Box MP 167, Mount Pleasant, Harare, Zimbabwe

**Keywords:** Chlorhexidine digluconate, ABC transporter, Nosocomial pathogens, Efflux pump inhibitor

## Abstract

**Background:**

Chlorhexidine digluconate (CHG) is used as a disinfectant. The emergence of pathogens resistant to the biocide raises health concern. Information on specific efflux mechanisms utilised by bacteria to confer reduced susceptibility to the biocide, may be used to develop ways of preventing the efflux of the biocide from nosocomial pathogens resulting in higher disinfection activity. The aim of the study was to evaluate the role of ATP-binding cassette transporters on the transport of CHG in bacteria.

**Methods:**

Clinical strains of *Pseudomonas aeruginosa*, *Staphylococcus aureus* and their respective laboratory strains ATCC 27853 and ATCC 9144 were used for susceptibility tests. The minimum inhibitory concentration (MIC) of CHG with or without an efflux pump inhibitor [reserpine or carbonyl cyanide *m*-chlorophenylhydrazone (CCCP)] was determined using the broth microdilution method. A spectrophotometric method to quantify the amount of chlorhexidine in a sample was developed, validated and used to quantify CHG within *P*. *aeruginosa* and *S*. *aureus* cells.

**Results:**

In the presence of reserpine, the MIC of CHG against the clinical strains of *P*. *aeruginosa* and *S*. *aureus* decreased from 6.3 to 3.2 µg/ml but showed no change against both ATCC isolates. The MIC of CHG in the presence of CCCP for both strains of *P*. *aeruginosa* remained unchanged but showed a reduction for both isolates of *S*. *aureus.* The suitability of the spectrophotometric method developed for quantifying the amount of CHG accumulated in microbial cells was validated and used successfully to quantify CHG accumulated within bacterial cells.

**Conclusion:**

The spectrophotometric determination of CHG within microbial cells may be used to quantify CHG in microbial cells. Only the clinical strain of *P*. *aeruginosa* showed significant efflux of CHG suggesting the participation of efflux transporters in the pumping out of CHG from this isolate. The use of efflux pump inhibitors together with the biocide may be explored to preventing the efflux of the biocide from *P*. *aeruginosa* resulting in order to increase disinfection activity.

## Background

Nosocomial pathogens are the main infectious agents of hospital acquired infections, resulting in significant morbidity and mortality within the healthcare facilities [1] as well as additional hospital costs. *Staphylococcus aureus*, *Enterococcus*, *Klebsiella* spp, *Acinetobacter* spp and *Pseudomonas aeruginosa* are some of the common nosocomial pathogens [2]. In a hospital, microbial pathogens may be found on equipment such as endotracheal tubes, catheters, soap dispensers and stethoscopes [3]. If disinfection is not sufficiently carried out, the contaminated equipment may become vectors of transmission of the nosocomial pathogen to the predisposed host. In an attempt to reduce nosocomial infections, preventative mechanisms have to be diligently effected to break down the triangle of the contagious agent, means of transmission and the predisposed host-the patient [4]. Disinfection and antisepsis using biocides are the main mode of action utilised in an effort to fight the growth of nosocomial pathogens. Peracetic acid, benzalkonium chloride, triclosan, sodium hypochlorite, and chlorhexidine gluconate are some of the commonly used biocides [5]. Chlorhexidine digluconate is considered to be the “gold standard biocide” showing broad spectrum activity and is used both as a disinfectant and antiseptic [1]. Both Gram-positive and Gram-negative bacteria show susceptibility to chlorhexidine and the biocide displays bactericidal as well as bacteriostatic activity depending on concentration [6]. The indiscriminate and incorrect use of biocides in agriculture, food production, human medicine and personal care products has resulted in the emergence of microorganisms showing resistance to biocides [7]. Validating the efficacy of disinfection is a vital but often difficult task. Minimal inhibitory concentrations (MICs) are usually used to study or compare the susceptibility of selected microbes towards a particular antimicrobial [8]. The determination of MICs involves using a range of dilutions of the biocide to define the concentration, which does not allow microbial growth for initial inocula of 1 × 10^6^ CFU/ml. A high MIC value depicts that a high concentration of a given antimicrobial is required to inhibit microbial growth, thus, the test isolate is highly resistant to that particular antimicrobial [1]. Due to the development of resistance of some microbes to biocides, nosocomial pathogens may not be completely eliminated, despite using antimicrobials in disinfecting hospital surfaces and equipment [4]. The continued proliferation of pathogens after disinfection may be attributed to the metabolism of the biocide, biofilm formation, changes in cell permeability or pumping out of the biocide from microbial cells by efflux pumps [9]. Efflux pumps are found in both prokaryotic and eukaryotic organisms. The efflux pumps are protein in nature and span the bacterial cell membrane, playing the role of transporting a particular substrate or an array of structurally similar compounds [10]. Microbial efflux pumps are divided into five main classes: MFS (major facilitator superfamily), MATE (multidrug and toxic compound extrusion), RND (resistance nodule division), SMR (small multidrug resistance) and ABC (ATP-binding cassette) [11]. Efflux pumps can be further categorised based on the driving source of energy utilised for the pumping out of the substrates. ABC pumps are primary transporters that use the energy of ATP binding and hydrolysis to export a variety of substrates across cellular membranes [12].

The action of efflux pumps may be blocked by efflux pump inhibitors (EPIs). Reserpine, verapamil, carbonyl cyanide *m*-chlorophenylhydrazone (CCCP) and chlorpromazine are some of the EPIs which can increase retention of some compounds within microbial cells [13]. CCCP affects the energy levels of bacterial membrane and is used to abolish the efflux of various molecules [14]. Reserpine inhibits efflux pumps by altering the generation of the proton-motive force required in the utility of MDR pumps. Reserpine has the ability to inhibit ABC pumps albeit in high concentrations [15]. To improve the efficacy of biocides showing reduced effectiveness, EPIs can be used in conjunction with biocides to act as inhibitors to enhance the efficacy of existing biocides. In this study, the role of ABC transporters in the transport of the biocide chlorhexidine digluconate was evaluated using CCCP and reserpine as efflux pump inhibitors.

## Methods

### Chemicals and materials

Chlorhexidine digluconate (CHG) aqueous standard 20% solution (C9394), ampicillin (A9518), reserpine (R0875), dimethyl sulphoxide (DMSO) (D5879), 3-(4,5-dimethylthiazolyl)-2, 5-diphenyltetrazolium bromide [MTT, thiazolyl blue] (M2128) and carbonyl cyanide *m*-chlorophenylhydrazone (CCCP) (C2759) used in the study were purchased from Sigma-Aldrich company (Germany). Glucose (G8270), Tryptic soy broth (TSB) (22092) and Tryptic soy agar (TSA) (22091) were also bought from Sigma-Aldrich Company (Germany). Distilled grade water was used for all the experiments.

### Bacterial strains

A total of four bacterial isolates were used in the study. The four isolates comprised a single clinical strain of *P. aeruginosa* and *S. aureus* along with their respective reference strains; ATCC 27853 and ATCC 9144. Clinical strains used were isolated from patients at Parirenyatwa Hospital (Department of Medical Microbiology, College of Health Sciences, University of Zimbabwe, Harare, Zimbabwe). The ATCC strains were acquired from the Microbiological Section in the Department of Biological Sciences at the University of Botswana (Gaborone, Botswana). All bacteria were kept as glycerol stocks at -35 °C and overnight cultures were used for each assay.

### Development of a chlorhexidine accumulation assay protocol

A chlorhexidine accumulation assay protocol was developed using the basic techniques of an accumulation assay established by Mortimer and Piddock [16]. A spectrophotometric method to quantify the CHG accumulated within bacterial cells was developed. A 2800 UNICO UV/VIS spectrophotometer (UNICO United Products and Instruments Inc., Dayton, United States) was used for quantifying the CHG. CHG absorbs optimally at 255 nm [17], thus, the measurement of CHG using a UV/VIS spectrophotometer was carried at a wavelength of 255 nm. The suitability of using the spectrophotometer was validated, by determining the optical density (OD) of a series of the standards of CHG prepared on the day. Standards of 0-12 µM CHG were prepared and analysed in duplicate. A calibration curve was plotted for the absorbances obtained from the series of standard using Graphpad™ version 5 for Windows (Graphpad™ Software Inc., San Diego, California, USA). The goodness-of-fit of linear regression, R^2^, obtained was used to determine the appropriateness of using a UV/VIS spectrophotometer for measuring the amount of CHG. Sub-inhibitory concentrations of chlorhexidine, CCCP and reserpine used in the accumulation assay were determined using half the MIC value (^1^/_2_MIC). A calibration curve was used to interpolate concentrations of chlorhexidine in samples in the accumulation assay.

### Determination of MIC

**Fig. 1:**
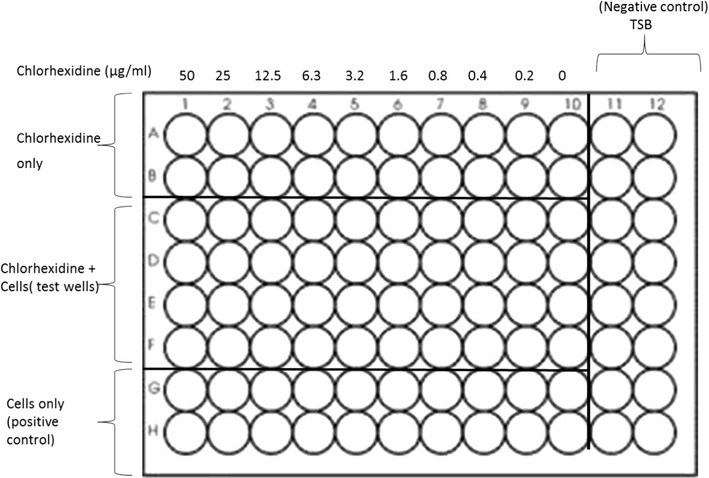
Example of a 96-well plate set up for determination of the MIC of chlorhexidine using the broth microdilution method. TSB (Tryptic Soy Broth) is the media used in the microdilution assay. Each test well contains 100 µl of defined chlorhexidine dilution and 100 µl of 2 × 10^6^ CFU/ml bacterial suspension. Negative control wells contain 200 µl media only to provide a turbidity control for reading end points. The positive control contained 100 µl of TSB and 100 µl of 2 × 10^6^ CFU/ml of the test isolate to give a final concentration of 10^6^ CFU/ml

Stock solutions of 5% CHG and 100 µg/ml ampicillin were prepared using sterile distilled water while the stocks of reserpine and CCCP were prepared in DMSO. All two-fold serial dilutions were made in TSB. The plate count method was used to determine the viable cell count of serially diluted overnight culture and appropriate dilutions made to reach 1 × 10^6^ CFU/ml. Microdilution tests of CHG, CCCP and reserpine, as recommended by the Clinical and Laboratory Standards Institute [18] were carried out in quadruplicate using 96-well microtitre plates. The general set-up of the 96-well microtitre plate is as shown in Fig. 1. Each test well was filled with 100 µl of defined chlorhexidine dilution and 100 µl of 2 × 10^6^ CFU/ml test isolate suspension. Wells for the positive control contained 100 µl of media and 100 µl of 2 × 10^6^ CFU/ml to give a final concentration of 10^6^ CFU/ml. After overnight incubation at 37 °C in a humidified atmosphere, bacterial growth was estimated as absorbance for turbidity using a Genios Pro microplate reader (Austria). A separate microtitre plate was set up to determine the MIC of ampicillin against all four isolates. The MIC of CHG in the presence of an EPI: CCCP or reserpine was also determined using sub-inhibitory concentrations (^1^/_2_ MIC value for the EPI calculated from MIC values initially obtained in the study). All microplates were incubated overnight at 37 °C in a Lab Companion incubator (Jeio Tech. Co. Ltd, Seoul, Korea) under a closed humidified atmosphere. Bacterial growth was determined as cell density using a Genios Pro microplate reader (Tecan Group Ltd, Grodig, Austria) to measure absorbance at 590 nm prior to and after incubation. The mean absorbance difference of varying concentrations of each bacterium in the presence CHG was compared to that of the media (TSB) that served as the negative control. The MIC was the minimum concentration of chlorhexidine that did not allow the growth of microbes. The MTT assay as outlined by Hansen et al., [19] with minor modifications, was utilised to visually observe bacterial viability. The MTT assay involved the addition of 30 µl of 1 mg/ml MTT to each well and reincubated for 1 h at 37 °C. The yellow colour of the tetrazolium salt would persist if cells were non-viable or be reduced by dehydrogenases and reductases in viable cells to a blue or purple colour. The reduced salt would form an insoluble precipitate, which was dissolved by the addition of 30 µl DMSO at the end of the incubation period. The MIC was determined as the minimum concentration of chlorhexidine in which no colour change in MTT was observed.

### Accumulation assay

The amount of CHG that accumulated in *P. aeruginosa* and *S. aureus* in the presence of standard efflux pump inhibitors, either CCCP or reserpine was studied using the protocol developed based on the procedure outlined by Mortimer and Piddock [16]. All tests were carried out in duplicate. A volume of 200 µl of an overnight culture was transferred into 200 ml TSB and incubated overnight at 37 °C with shaking (120 rpm). Cells were collected from the broth by centrifuging at 3000 rpm for 15 min using a Rotafix Centrifuge (Taufkirchen, Germany), washed two times using phosphate buffer solution (PBS), pH 7.4. The washed cells were suspended in PBS and centrifuged for 15 min. The pellet was resuspended in PBS containing sodium azide to give a final cell concentration of 40 mg/ml. A final concentration of ^1^/_2_MIC of CHG was added to the cells. The mixture was incubated at 37 °C for 30 min with shaking (120 rpm). Cells were collected by centrifuging at 4000 rpm for 15 min and distributed into 5 ml aliquots of tubes A to D. Tube A was the negative control containing no glucose thus no source of energy. Tube B was the positive control containing 1.0 M glucose being the source of ATP. Tubes C contained 1.0 M glucose and a final concentration of ^1^/_2_MIC of CCCP (calculated from the MIC value CCCP). Tube D contained 1.0 M glucose and a final concentration of ^1^/_2_MIC of reserpine. All tubes were incubated at 37 °C with agitation (120 rpm) for 1 hour in a Lab Companion incubator. Cells were collected by centrifuging at 4000 rpm for 15 min and the supernatant stored for CHG efflux quantification. The pellet obtained from each tube was resuspended in 0.1 M glycine–HCl pH 3, was mixed using a vortex mixer and incubated overnight to allow cell lysis. The tubes with lysed cells were centrifuged at 4000 rpm for 15 min and the supernatant retained for quantification of CHG. The quantification of CHG of the supernatants was performed using a 2800 UNICO UV/VIS spectrophotometer (UNICO United Products and Instruments Inc., Dayton, United States) at a wavelength of 255 nm. Sample concentrations were interpolated from a standard curve initially plotted using known standards of CHG. Sample quantities obtained from interpolated values were expressed as percentage chlorhexidine accumulated (% CHX accumulated) in relation to possible accumulation in the presence of energy in the form of ATP from glucose, calculated as:

$$\frac{{[{\text{chlorhexidine}}_{\text{sample}} ]}}{{[{\text{chlorhexidine}}_{\text{control}} ]}} \times 100$$

where, [chlorhexidine_sample_] is concentration of chlorhexidine under different treatments and [Chlorhexidine_control_] is concentration of chlorhexidine of control (glucose only).

### Statistical analyses

Graphpad™ for Windows Version 5 (Graphpad™ Software Inc., San Diego, California, USA) was used for statistical analyses of the results. One way analysis of variance (ANOVA) and a Dunnet post-test was used to compare results obtained for the positive control against that of test samples a P value of 0.05 or less were considered significant.

## Results

### Quantification of chlorhexidine using a UV/VIS analyses and the effects of standard EPIs on the MIC of CHG

**Fig. 2:**
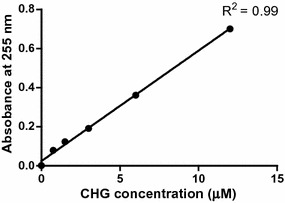
Absorbance of CHG at 255 nm as a function of concentration. The *graph* shows that CHG obeys the Beer-Lambert Law at concentrations between 0 and 12 µM

There was a linear correlation between absorbance and concentration of CHG at 255 nm with a value of R^2^ of 0.99 as shown in Fig. 2. Thus, chlorhexidine concentrations of test samples could be interpolated from the calibration curve.

MICs of chlorhexidine against the clinical and ATCC strains of *P. aeruginosa* and *S. aureus* obtained in the absence and presence of CCCP or reserpine are as shown in Table 1.

**Table 1:** MICs of chlorhexidine against the clinical and ATCC strains of *P. aeruginosa* and *S. aureus* obtained in the absence and presence of CCCP or reserpine

Determined MIC	*P. aeruginosa* clinical strain (µg/ml)	*P. aeruginosa* clinical strain	*S. aureus* clinical strain	*S. aureus* ATCC strain
Chlorhexidine only	6.3	6.3	6.3	6.3
Chlorhexidine + CCCP	6.3	6.3	*<0.1*	*<0.1*
Chlorhexidine + reserpine	*3.2*	*3.2*	*3.2*	6.3

Italics indicates a decrease in the MIC of chlorhexidine in the presence of an EPI

The MIC values of CHG remained unchanged (Table 1) for both the clinical and laboratory isolates of *P. aeruginosa* (6.3 and 3.2 µg/ml respectively) in the presence of CCCP. A two-fold reduction in MIC was observed when CHG was used together with 63 µg/ml reserpine against the clinical strain of *P. aeruginosa*. No change in the MIC of CHG against the laboratory isolate of *P. aeruginosa* in the presence of reserpine was noted. The clinical and ATCC isolates of *S. aureus* showed reduced susceptibility to CHG when used in combination with CCCP (Table 1). The MIC of CHG for the two *S. aureus* strains was below 0.1 µg/ml, which is lower than 6.3 µg/ml obtained when CHG was used on its own for both strains. The MIC of CHG combined with reserpine against the clinical strain of *S. aureus* was reduced two-fold (from 6.3 to 3.2 µg/ml). However, the MIC of CHG against the laboratory strain remained at the same level of 6.3 µg/ml despite the presence of the efflux inhibitor reserpine.

### Accumulation of CHG in *P. aeruginosa* and *S. aureus*

**Fig. 3:**
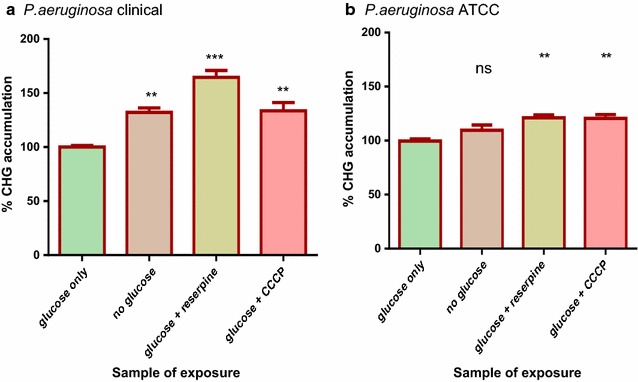
CHG accumulation assays for the clinical (**a**) and ATCC (**b**) strains of *P. aeruginosa*. Cells exposed to glucose served as the positive control where 100% accumulation occurred. The % CHG accumulation for rest of the sample treatments was in comparison to the positive control. The* error bars* show the standard deviation from the mean of two samples read twice. The difference between the control and other sample treatments was tested at 95% confidence interval. The* asterisks* indicate a significant difference from the control with **p < 0.01, ***p < 0.001 and ns shows no significant difference from the control

**Fig. 4:**
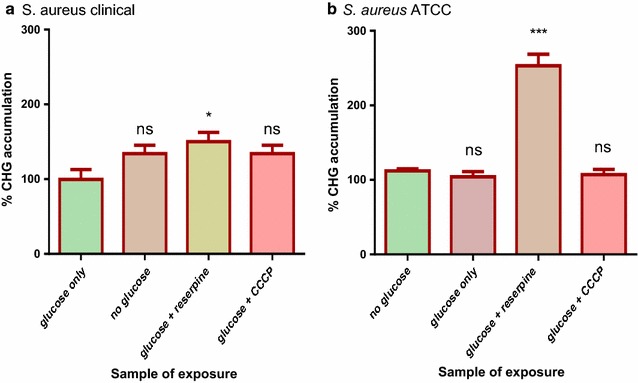
CHG accumulation assays for the clinical (**a**) and ATCC (**b**) strains of *S. aureus*. Cells exposed to glucose served as the positive control where 100% accumulation occurred. The % CHG accumulation for rest of the sample treatments was in comparison to the positive control. The* error bars* show the standard deviation from the mean of two samples read twice. The difference between the control and other sample treatments was tested at 95% confidence interval. The* asterisks* indicate a significant difference from the control with *p < 0.05, ***p < 0.001 and ns shows no significant difference from the control

Concentrations of chlorhexidine accumulated under different treatment conditions interpolated from the standard curve were expressed as percentages shown in Fig. 3. One way ANOVA and Dunnett’s test was used to compare the different test treatments against the control to determine whether there was statistical differences in chlorhexidine accumulated under different treatment conditions. The cells from the tube containing glucose had the lowest amount of accumulated CHG showing that CHG was actively pumped out of *P. aeruginosa* resulting in the net low CHG accumulated in both the clinical and laboratory strains (Fig. 3). When compared to the cells exposed to glucose, cells without glucose had moderately significant higher CHG accumulated within the clinical strain cells. There were no significant differences in the amount of CHG accumulated by cells without glucose and cells exposed to glucose for the ATCC strain. The accumulation of CHG in cells in the presence of both CCCP and reserpine was significantly higher for both the clinical and ATCC strains. Reserpine resulted in a higher accumulation of chlorhexidine than CCCP within the clinical strain cells. The clinical strain showed 22.5% more efflux activity than the laboratory strain as cells without glucose. The cells exposed to inhibitors had significantly higher amounts of accumulated CHG. Both strains of *S. aureus* cells exposed to glucose contained amounts of CHG which was not significantly different from cells incubated with no glucose (Fig. 4). Efflux activity for both the clinical and ATCC strain of *S. aureus* was not significantly different.

## Discussion

Resistance to biocides occurs mainly as a result of target alteration, reduced accumulation due to decreased permeability, inactivation/modification and increased efflux. Efflux pumps are prominent in terms of drug extrusion underlying their roles in multidrug resistance [20]. Bacterial ABC efflux pumps play a vital role in cell viability, pathogenicity as well as virulence [21]. Bacterial pathogenesis can be enhanced by ABC transporters by the extrusion of toxins, disinfectants and drugs from the cell, rendering them ineffective against the bacterium [22].

The antimicrobial properties; efficacy and disinfecting efficiency of chlorhexidine have been documented [23, 24], but very limited information is available for the possible emergence of resistance due to lowered concentration in the bacterial cells. The available methods make use High Performance liquid chromatography HPLC [25] to quantify the amount of chlorhexidine in samples such as milk and saliva. An alternative and simple method which makes use of a 2800 UV/VIS spectrophotometer instead of an HPLC was developed in this study. Chlorhexidine absorbance was measured at 255 nm because the biocide absorbs maximally at 255 nm [15]. The use of the UV/VIS spectrophotometer was validated by generating a calibration curve using a series of chlorhexidine concentrations. The R^2^ value of 0.99 (Fig. 2) obtained confirmed that at 255 nm there was a significant linear relationship between absorbance and the concentration of chlorhexidine. Therefore, the UV/VIS spectrophotometer could be used to quantify the amount of CHG in the accumulation assay. To ensure that growth of cells used in the accumulation assay was not inhibited, sub-inhibitory (^1^/_2_MIC) concentrations of chlorhexidine, CCCP and reserpine were used from MIC obtained in the study using the broth microdilution.

The MICs of chlorhexidine in the absence and presence of EPIs against the clinical and ATCC strains of *P. aeruginosa* and *S. aureus* were compared, in order to determine if the presence of an EPI reduces the MIC value of chlorhexidine. No difference in MIC values of chlorhexidine was noted for chlorhexidine in combination with CCCP against the clinical and ATCC strains of *P. aeruginosa*. In another study, this poor efficacy of CCCP towards *P. aeruginosa* was attributed to a difference in sequences of AcrB and MexB which may induce some changes in the functional organisation of the efflux pumps [26]. However, there was a reduction in the MIC of chlorhexidine in the presence of the MDR inhibitor reserpine, which was evidence of inhibitory activity also reported by Omoregie et al., [27]. The reduction in the MIC value in the presence of reserpine was also found to give an inhibitory effect on the clinical strain of *P. aeruginosa* efflux pumps [27]. The reduction in MIC values of chlorhexidine in the presence of an EPI (Table 1) obtained for the clinical strain of *P. aeruginosa*, the clinical and ATCC strains of *S. aureus* could only show the presence of efflux activity without bringing out the type of efflux pump involved. An accumulation assay in the presence and absence of glucose an energy source was carried out for all isolates in order to evaluate the role of ABC transporters in the pumping out of CHG from bacterial cells.

Since ABC transporters are the only type of efflux pumps that use ATP as their primary source of energy [10] any differences observed in the amount of chlorhexidine accumulated by cells in the presence of glucose could be attributed to the activity of ABC transporters. The clinical strain of *P. aeruginosa* showed a significant reduction in levels of chlorhexidine accumulated in presence of glucose than in the absence of glucose (Fig. 3). This evidence may suggest active efflux of chlorhexidine by ABC pumps which may translate to higher virulence. In a comparative study, the clinical strain of *P. aeruginosa* was reported to have upregulated proteins linked with virulence and increased pathogenesis which indicated that the clinical strain is more virulent than the laboratory strain [15]. Cells of the clinical strain of *P. aeruginosa* incubated in the presence of glucose plus reserpine showed significantly higher levels of chlorhexidine accumulated than cells incubated in the presence of glucose only. This showed that reserpine was inhibiting efflux pump activity of the ABC transporters. Concentrations of up to 100 µg/ml reserpine have been reported to give an inhibitory effect on *P. aeruginosa* efflux pumps [27]. Both strains of *S*. *aureus* showed no significant reduction in the levels of chlorhexidine accumulated in presence of glucose, evidence that there was no active efflux of chlorhexidine by ABC transporters. Efflux activity in *S*. *aureus* suggested by the reduction in MIC values of chlorhexidine in the presence of an EPI (Table 1) may be attributed to other types of efflux pumps. An example of an efflux pump type that can pump out chlorhexidine without the use ATP as a primary source of energy is the major facilitator superfamily (MFS) [11]. MFS efflux pumps involved in the uniport, antiport and symport of a variety of compounds across the cell have been identified in *S. aureus* [28]. The findings of this study suggest that ABC transporters may play a role in the efflux of CHG from the clinical strain of *P. aeruginosa*, since there was a net decrease in the accumulation of CHG within the bacteria. A low concentration of the biocide allows the bacteria to adapt to its new environment [29] potentiating the Gram-negative opportunistic pathogen to survive disinfection and cause nosocomial infections of the urinary tract, respiratory system and gastrointestinal tract [30]. On the contrary, the role of ABC transporters in the efflux of CHG from *S. aureus* was not significant, resulting in a net high accumulation of CHG within the Gram-positive bacteria. This concurs with the findings in previous studies that showed that Gram-negative bacteria generally tend to be less sensitive to many antimicrobials than Gram-positive bacteria [11]. Traditionally, the reduced sensitivity of Gram-negative microbes to antimicrobial has been accredited to the “permeability barrier” conferred by the bacterial cell envelope, which limits uptake into the cell [31].

## Conclusion

The development and validation of an assay to quantify the amount of chlorhexidine accumulated in bacterial cells was achieved. The role of ABC transporters in the efflux of chlorhexidine from clinical and ATCC strains of *S*. *aureus* as well as ATCC strains of *P. aeruginosa* was not evident. ABC transporters play a role in the efflux of chlorhexidine from the clinical strain of *P. aeruginosa*. The effectiveness of CHG against the bacteria may be lowered, thus, increasing the possibility of the bacteria to flourish in hospital environments.

## Abbreviations

CHG: chlorhexidine digluconate
ABC: ATP-binding cassette
ATCC: American type control culture
MIC: minimum inhibitory concentration
CCCP: carbonyl cyanide *m*-chlorophenylhydrazone
EPI: efflux pump inhibitor
CFU: colony forming units
DMSO: dimethyl sulfoxide
MTT: 3-(4,5-dimethylthiazolyl)-2,5-diphenyltetrazolium
TSB: tryptic soy broth
TSA: tryptic soy agar
PBS: phosphate buffered saline
MFS: multidrug facilitator superfamily
MATE: multidrug and toxic compound extrusion
RND: resistance nodule division
SMR: small multidrug resistance
